# Therapeutic targeting of extracellular DNA improves the outcome of intestinal ischemic reperfusion injury in neonatal rats

**DOI:** 10.1038/s41598-017-15807-6

**Published:** 2017-11-13

**Authors:** Michael Boettcher, Georg Eschenburg, Stefan Mietzsch, Miguel Jiménez-Alcázar, Michaela Klinke, Deirdre Vincent, Bastian Tiemann, Robert Bergholz, Konrad Reinshagen, Tobias A. Fuchs

**Affiliations:** 1 Department of Pediatric Surgery, University Medical Center Hamburg-Eppendorf, Martinistrasse 52, 20246 Hamburg, Germany; 2 Institute of Clinical Chemistry and Laboratory Medicine, University Medical Center Hamburg-Eppendorf, Martinistrasse 52, 20246 Hamburg, Germany; 3 Department of Experimental Animal Research, University Medical Center Hamburg-Eppendorf, Martinistrasse 52, 20246 Hamburg, Germany

## Abstract

Thrombosis and inflammation cooperate in the development of intestinal infarction. Recent studies suggest that extracellular DNA released by damaged cells or neutrophils in form of extracellular traps (NETs) contributes to organ damage in experimental models of ischemia-reperfusion injury. Here we compared the therapeutic effects of targeting fibrin or extracellular DNA in intestinal infarction after midgut volvulus in rats. Following iatrogenic midgut volvulus induction for 3 hours, we treated animals with a combination of tissue plasminogen activator (tPA) and low molecular weight heparin (LMWH) to target fibrin or with DNase1 to degrade extracellular DNA. The therapeutic effects of tPA/LMWH and DNase1 were analyzed after 7 days. We observed that both therapeutic interventions ameliorated tissue injury, apoptosis, and oxidative stress in the intestine. DNase1, but not tPA/LMWH, reduced intestinal neutrophil infiltration and histone-myeloperoxidase-complexes, a surrogate marker of NETs, in circulation. Importantly, tPA/LMWH, but not DNase1, interfered with hemostasis as evidenced by a prolonged tail bleeding time. In conclusion, our data suggest that the therapeutic targeting of fibrin and extracellular DNA improves the outcome of midgut volvulus in rats. DNase1 therapy reduces the inflammatory response including NETs without increasing the risk of bleeding. Thus, targeting of extracellular DNA may provide a safe therapy for patients with intestinal infarction in future.

## Introduction

Midgut volvulus refers to a condition in which the midgut twists around the axis of the superior mesenteric artery, a disorder with a reported annual incidence of 1.7–5.7:100000^1^. It is most likely to occur in neonates and young infants, and is often related to malrotation^2,3^. Midgut volvulus leads to considerable morbidity and mortality (in neonates 23–38%)^4^ and late diagnosis of the condition requires extensive surgical removal of affected bowel. This ultimately results in intestinal failure causing substantial morbidity, such as liver failure due long term parenteral nutrition^5^.

Ischemia and reperfusion (IR) of the intestine during midgut volvulus induces severe oxidative stress and inflammation. IR injury is particularly distinctive in neonates as compensatory mechanisms are immature and the inflammatory reactions are more pronounced^6,7^. Recently, thrombosis has been found to play a major role in the development of intestinal infarction^4,8^. Under physiological conditions, procoagulant and anticoagulant mechanisms are balanced, thus preventing intravascular coagulation. However, tissue ischemia induces a pro-thrombotic state leading to vascular dysfunction, ultimately causing thrombosis^4,9,10^. This results in a “no-reflow” phenomenon, characterized by absent reperfusion in microvasculature during macrovascular reperfusion of ischemic tissue^4^. A recent case report showed that systemic thrombolysis, as treatment for midgut volvulus in neonates, caused excellent gut viability in two unfavorable cases^11^, supporting the notion that thrombosis contributes to the volvulus pathomechanism.

Thrombosis closely cooperates with inflammation^12^. Neutrophils, the primary leukocyte in acute inflammation, represent an important factor in the protracted inflammatory response, as well as severity, associated with intestinal IR injury^13^. Neutrophils may contribute to IR injury by forming extracellular traps (NETs), webs of DNA-filaments that are decorated with toxic proteins^14^. Neutrophils release NETs upon microbial detection to immobilize and kill pathogens^14,15^. However, an uncontrolled or excessive release of NETs is associated with decreased microvascular perfusion and tissue damage^16,17^.

NETs released within the vasculature may cause platelet adhesion and activation of the extrinsic and intrinsic coagulation cascade^18–20^. Moreover, therapeutic infusions of DNase1 to degrade extracellular DNA improved the outcome of ischemic stroke and myocardial infarction in mice^21,22^, indicating that NETs may play a significant role in the pathogenesis of IR injury.

Here, we hypothesized that the therapeutic applications of DNase1 may provide an alternative treatment strategy to reduce intestinal reperfusion injury in midgut volvulus. We therefore compared DNase1 therapy to a conventional anti-thrombotic therapy, comprised of low molecular weight heparin and tissue plasminogen activator (tPA).

## Material and Methods

### Study design

The study was approved by the Hamburg State Administration for animal research (Protocol number: 88/14). Animals experiments were performed in accordance with the German guidelines for laboratory animal care and procedures (Tierschutzgesetz). A total of 61 female Wistar rats (Charles River, Wilmington, USA) aged two weeks were utilized for the experiment.

### Animal Procedures

For better standardization one surgeon performed all operations. Anesthesia was induced with 5% isoflurane (Baxter, Unterschleißheim, Germany) and maintained during the surgical interventions using 2–3% isoflurane delivered through a facemask. Preoperative antisepsis was performed with phenoxyethanol. Moreover, all rats received 0.02 mg/kg s.c. buprenorphine (Reckitt Benckiser, Mannheim, Germany) for analgesia and 10 mg/kg s.c. enrofloxacin subcutaneously (Bayer, Leverkusen, Germany) as a prophylactic antibiotic therapy. At the beginning of the experiment 0.2 ml of blood was collected from each subject via retro-orbital sinus sampling of the right side.

The rats were placed in a dorsal decubitus position while a median laparotomy was performed. The small intestine was exposed and twisted 360°. The torsion was fixed in this position through two points with a 6 × 0 Prolene suture (Ethicon, Norderstedt, Germany). The abdomen was closed with a running suture using 5 × 0 Prolene. After three hours, the abdomen was reopened and the intestine was detorsed to its original physiological position. In all animals’ signs of reperfusion were observed. Finally, the abdomen was closed with simple interrupted sutures using 5 × 0 Prolene.

For the therapeutic intervention, we used low-molecular weight heparin (LMWH; Enoxaparin, Clexane, Sanofi-Aventis, Germany), tissue-plasminogen activator (tPA; Alteplase, Activase, Boehringer Ingelheim, Germany), and DNase1 (Pulmozyme, Roche, Mannheim, Germany). The rats were randomly divided into three groups receiving following treatments:

1. Control group: a single bolus of 8 mg/kg body weight inactivated DNase1 i.p. once daily for 48 hours.
2. LWMH/tPA group: a single bolus of 0.9 mg/kg body weight alteplase s.c. and 200 IU/kg body weight of enoxaparin s.c. once daily for 48 hours.
3. DNase1 group: a single bolus of 8 mg/kg body weight active DNase1 i.p. once daily for 48 hours.

Inactivation of DNase1 was reached by heating Pulmozyme to 100 degrees Celsius in a hot water bath for one hour. Dosages for enoxaparin, alteplase and DNase1 were deduced from thrombolysis protocols for humans and rats using the dosage translation technic of Reagan-Shaw *et al*.^23–27^. Application of highly concentrated DNase1 dissolves NETs *in vivo* ^15^. The medication was administered immediately after detorsion and closure of the abdominal incision and re-administered once daily for two days. After discontinuation of anesthesia, all animals were housed in the animal facility and received 10 mg/ml tramadol mixed with their drinking water for pain control. On day seven all rats were anaesthetized using isoflurane as described above. Re-laparotomy followed by resection of four areas of affected small intestine, as well as two areas proximal to the torsion, was performed. Additionally, 1–2 ml blood was withdrawn via intracardial puncture. Finally, all animals were euthanatized through intracardial injection of 40 mg/kg body weight thiopenthal (Inresa, Freiburg, Germany).

### Tissue preparation and evaluation

The excised intestine was weighed and divided for further processing. A small sample of the intestine was flash frozen using liquid nitrogen and stored at −80 °C, while another slice was placed in RNAlater solution *(*Life Technologies, Darmstadt, Germany), stored for 24 h at 4 °C, and then frozen at −80 °C. The remaining tissue was fixed in Bouin’s solution to preserve nuclei and chromosomes in meiosis^28^. These samples were then embedded in paraffin, cut in 3 µm thick sections and stained using hematoxylin and eosin (H&E), as well as periodic acid–Schiff (PAS). Analysis was conducted by a pathologist, blinded to the study, who examined the intestine using light microscopy.

The Chiu score was applied to evaluate intestinal damage in four different areas of affected (twisted) and two different areas of unaffected small intestine (areas proximal to the torsion)^29^:

- grade 0 (normal mucosal villi)
- grade 1 (subepithelial space at the villus tip)
- grade 2 (extension of the subepithelial space with moderate lifting of the epithelial layer)
- grade 3 (massive epithelial lifting along the villus sides)
- grade 4 (denuded villi with exposed dilated capillaries)
- grade 5 (digestion and disintegration of the lamina propria, hemorrhage, and ulceration)

### Immunohistochemistry

Tissue sections were analyzed for: (1) apoptosis using a rabbit anti-cleaved caspase 3 (Asp175) antibody (Cell Signaling, #9661); (2) neutrophil recruitment using a rabbit anti-neutrophil elastase antibody (Abcam, ab68672). Subsequently samples were incubated with a biotinylated anti-rabbit antibody and visualized using the HRP-AEC system (R&D, CTS006) as per manufacturer’s instructions. Isotype control antibodies were used as a negative control for each immunohistochemistry staining.

### TUNEL assay

A TUNEL assay to detect DNA fragmentation in cell nuclei (a marker for apoptosis in intestinal tissue) was performed using an *In Situ* Cell Death Detection Kit (Roche, Mannheim, Germany). Stained nuclei in 10 cross-sections per intestinal tissue section were assessed in a standardized mode using the Nucleus counter plugin by ImageJ 2.0.0 (Fiji distribution) and averaged for each subject. The results are expressed as apoptotic cells per cross-section.

### Malondialdehyde (MDA) Assay

Tissue MDA activity, a marker for lipid peroxidation, was assessed using the Lipid Peroxidation (MDA) Assay Kit (Sigma-Aldrich, MO, USA) according to manufacturer’s instructions. The results are expressed as nmol/g tissue.

### Glutathione Peroxidase (GPx) Assay

Tissue GPx activity, a marker for systemic antioxidant status, was measured using a Glutathione Peroxidase Assay Kit (Cayman, MI, USA) according to manufacturer’s instructions. The results are expressed as nmol/min/ml.

### Myeloperoxidase (MPO)

Tissue MPO activity, a marker for neutrophil infiltration, was quantified using a Rat MPO ELISA Kit (Hycult biotech, Uden, The Netherlands) according to manufacturer’s instructions. The results are expressed as ng/mg tissue.

### MPO-Histone-ELISA

Complexes of MPO and histones in serum, a surrogate marker of NETs, were quantified using a MPO-Histone ELISA Kit (Cayman, MI, USA) according to manufacturer’s instructions. The results are expressed as ng/ml.

### Blood analysis

Blood samples were taken immediately prior to the intervention, and at two, four, six hours, and in some animals at day seven post intervention. Upon sample collection, they were centrifuged for 10 minutes at 2000 × g and stored at −80 °C until further analysis.

### Plasma analysis

Plasma was collected and analyzed for cell-free DNA (cfDNA) quantified based on a previously described method^30^. D-dimers and thrombin-antithrombin complexes (TAT) were measured using ELISA kits, as recommended by the manufacturer (Cusabio, CA, USA). TAT and D-dimers are both markers of hypercoagulability: (1) TAT levels are enhanced by the generation of thrombin, while (2) D-dimers, fibrin degradation products, are markers of fibrinolysis in response to the activation of coagulation. Normal thresholds in rats of D-dimers and TAT are 500 ng/ml and 2  ng/ml, respectively^31,32^.

### Tail bleeding assay

In 14 animals, a tail bleeding assay was performed. One hour after detorsion and thrombolysis (LMWH/tPA) or active (DNase1) or inactivated DNase1 (Control) treatment, animals were placed in prone position and a 1 cm segment of the tail was amputated at the distal end using a scalpel. The tail was immediately immersed in an Eppendorf tube containing isotonic saline, which was pre-warmed in a water bath to 37 °C. The position of the tail was vertical with the tip positioned about 2 cm below the body horizon. Each animal was monitored for 20 minutes to detect any re-bleeding, even if bleeding ceased. Bleeding time was determined using a stopwatch. If bleeding on/off cycles occurred, the sum of bleeding times within the 20-minute period was used. The experiment was terminated at the end of 20 minutes to avoid lethality during the experiment.

### Statistics

All data were analyzed using SPSS Statistics 23 (IBM, NY, USA) and GraphPad Prism 6 (GraphPad, CA, USA). A pre-power study calculation was performed using G*Power 3.0, while the power was deducted from a previous study examining testicular torsion in rats^33^. Differences between groups were calculated using ANOVA with Turkey’s post hoc test. Data are presented as mean ± standard deviation (SD). For ordinal data, differences were calculated with Kruskal-Wallis test. To assess post-torsion gains, a repeated measures ANOVA was applied. For association between factors, Spearman’s Rho was utilized. For all tests, the level of significance was set at <0.05.

## Results

**Figure 1:**
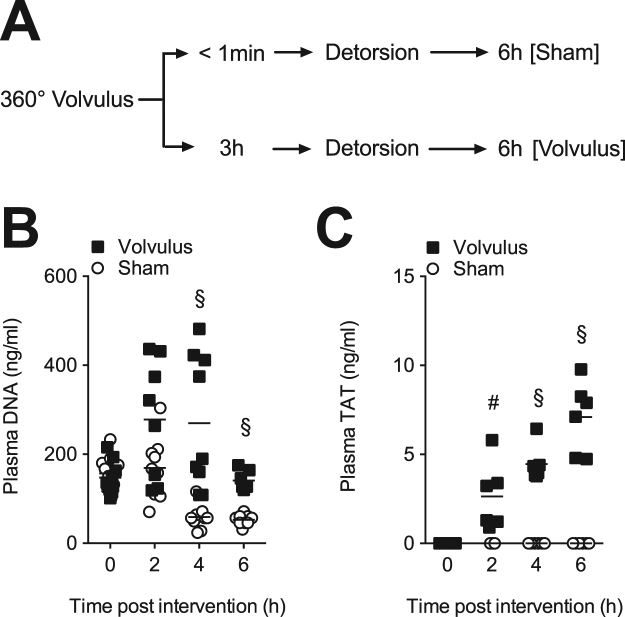
Midgut volvulus generates extracellular DNA and fibrin. (**A**) Experimental design. Rats were subjected to 360° volvulus for <1 min (Sham; N = 10) or 3 hours (Volvulus: N = 9). Immediately after and 2, 4 and 6 hour after detorsion, cfDNA and TAT levels were measured. (**B**) CfDNA is released by neutrophils in form of NETs, but can also be emitted from injured cells. In this study, cfDNA levels were significantly increased at two, four and six hours post volvulus as compared to sham operated animals, indicating severe cell injury in the volvulus group. (**C**) TAT is a marker of hypercoagulability. TAT levels were significantly increased at two, four and six hours post volvulus compared to shams. Data shown as Mean. Statistics: repeated t-test.

To determine whether extracellular DNA and fibrin are being generated following midgut volvolus in rats, we collected plasma after two, four, and six hours of volvulus as well as from sham-operated controls (Fig. 1A). We observed a significant increase in circulating extracellular DNA within 4 hours post midgut volvulus, when compared to controls (Fig. 1B). Furthermore, TAT levels, a marker for thrombin generation, steadily and significantly increased after mid gut volvulus, but not in controls (Fig. 1C). Levels of D-dimers, a fibrin degradation product, remained below the threshold in the 6-hour time period in both groups (not shown), which is in line with a previous study^31^.

**Figure 2:**
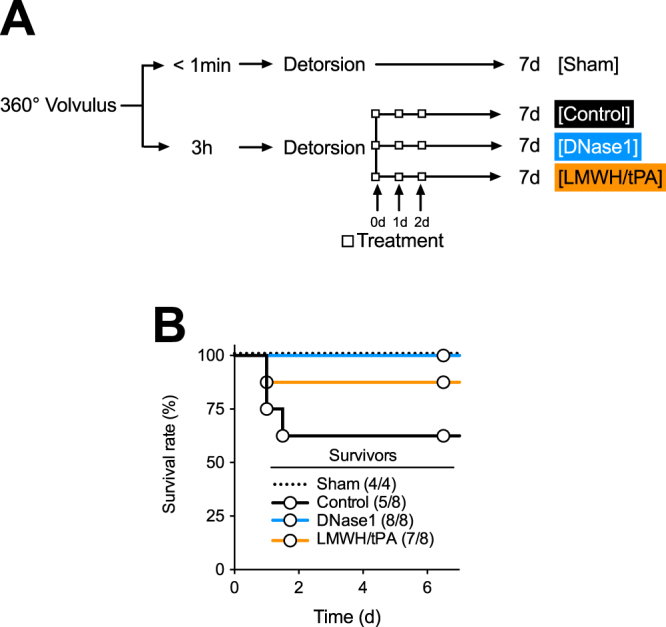
Experimental design and treatment strategy of midgut volvolus. (**A**) Experimental design. Rats were subjected to 360° volvulus for <1 min (Sham; N = 4) or 3 hours. Rats received inactivated DNase1 (Control; N = 8), active DNase1 (DNase1, N = 8), or a combination of LMWH and tPA (LMWH/tPA, N = 8) immediately after detorsion of the intestine. (**B**) Survival of the rats after volvulus or sham and treatment. Three rats in the control group and one rat in the LMWH/tPA group did not survive volvulus.

To determine the effect of DNase1- and LMWH/tPA therapy on the outcome of midgut volvulus, we applied the therapy immediately after detorsion of the intestine and every 24 hours for 2 days and analyzed tissue damage, apoptosis and oxidative stress in the intestine after 7 days (Fig. 2A). Of 28 rats that were included in this experiment, four rats did not survive midgut volvulus; 3 rats in the control group and one rat in the thrombolysis group (Fig. 2B).

**Figure 3:**
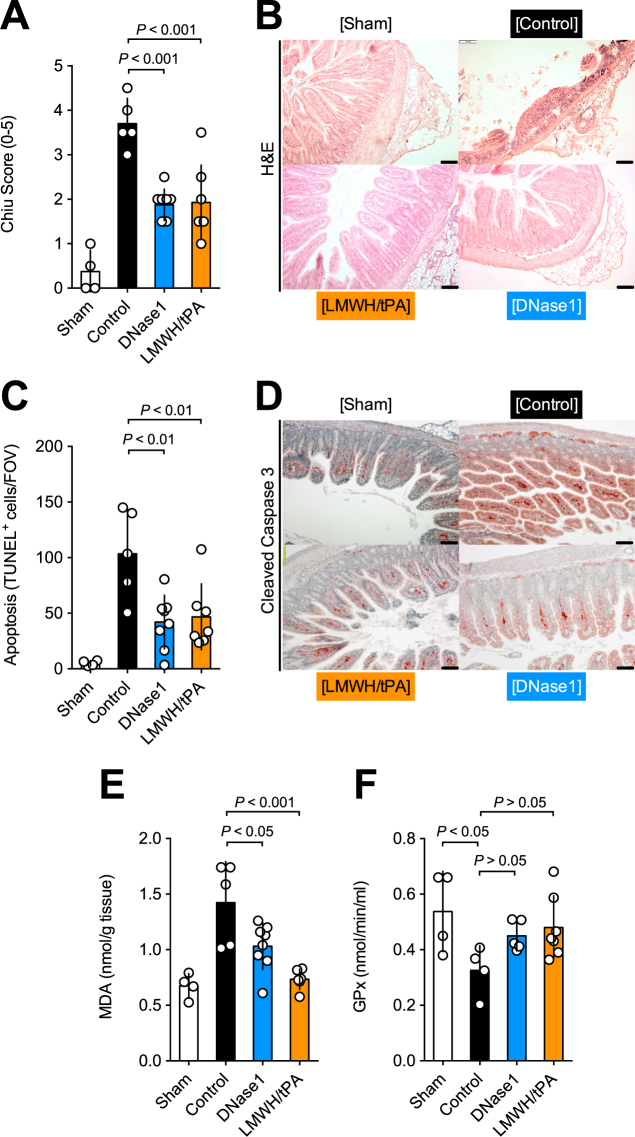
LMWH/tPA- and DNase1-treatment ameliorate tissue damage, apoptosis, and oxidative stress after midgut volvolus. (**A**) Chiu Score to assess intestinal damage. (**B**) HE staining: treatment with DNase1 or LMWH/tPA showed a significant improvement of gut viability after iatrogenic volvulus compared to controls. (**C**) Quantification of apoptotic cells per cross section detected by TUNEL. (**D**) Detection of apoptotic cells in tissue by staining for cleaved caspase 3 (brown). (**E**,**F**) MDA (oxidative stress) and GPx (antioxidative capacity) levels in tissue extracts. Treatment with either LMWH/tPA or DNase1 reduced oxidative stress relative to control animals, as demonstrated by a significant increase in MDA activity and a decrease in GPx activity in the controls as compared to treated animals. Data shown as Mean ± SD. Statistics: Kruskal-Wallis test, ANOVA.

We quantified the intestinal damage by histology using the Chiu score. Both treatment groups showed a significant reduction of mucosal injury as compared to controls (Fig. 3A,B). In addition, analysis of DNA-fragmentation by TUNEL-staining as well as immunostaining for activated caspase 3 revealed a significant reduction of apoptotic cells within the intestine in respone to either treatment (Fig. 3C,D). Furthermore, we determined MDA and GPx to assess lipid peroxidation and oxidative stress in intestinal tissue, respectively. The MDA levels indicated a significant reduction of oxidative stress upon DNase1 or LMWH/tPA (Fig. 3E). In addition, GPx was less reduced in rats treated with DNase1 or LMWH/tPA than in controls (Fig. 3F). Collectively, the data suggest that therapeutic targeting of thrombosis and extracellular DNA preserves the integrity of intestinal tissue post midgut volvulus.

**Figure 4:**
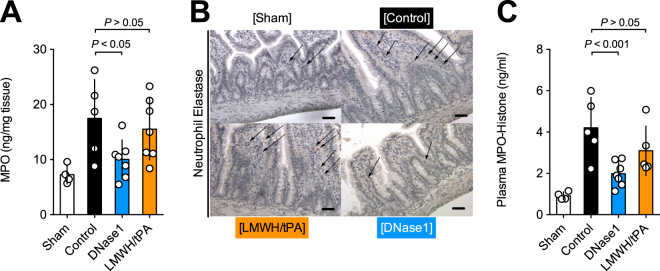
DNase1, but not LMWH/tPA, neutrophil infiltration and NET-markers after midgut volvulus. (**A**) Quantification of MPO in intestinal tissue extractions to assess neutrophil infiltration. (**B**) Detection of intestinal neutrophils by staining for neutrophil elastase (NE). (**C**) Quantification of the NET-markers MPO-histone-complex in plasma. DNase1, but not LMWH/tPA treatment significantly reduces the number of neutrophils and NET-markers. Data shown as Mean ± SD. Statistics: ANOVA.

We now quantified neutrophils in tissue and NETs in circulation to further characterize the tissue protective effect of DNase1 and LMWH/tPA. To assess neutrophil infiltration into intestinal tissue, we quantified using MPO in tissue extracts (Fig. 4A) and stained the intestine for NE (Fig. 4B). Interestingly, DNase1-treatment, but not LMWH/tPA, significantly reduced the levels of MPO and NE-positive cells in the intestine. Next, we quantified NETs using MPO-histones-complexes in plasma (Fig. 4C) as surrogate marker. MPO-histones levels were significantly lower with DNase1 treatment than in controls, indicating a reduction of NET-formation (Fig. 4C). Interestingly, LMWH/tPA did not reduce MPO-histone levels. Taken together, these data indicate that DNase1, but not LMWH/tPA, reduces inflammation in the intestine after midgut volvulus.

**Figure 5:**
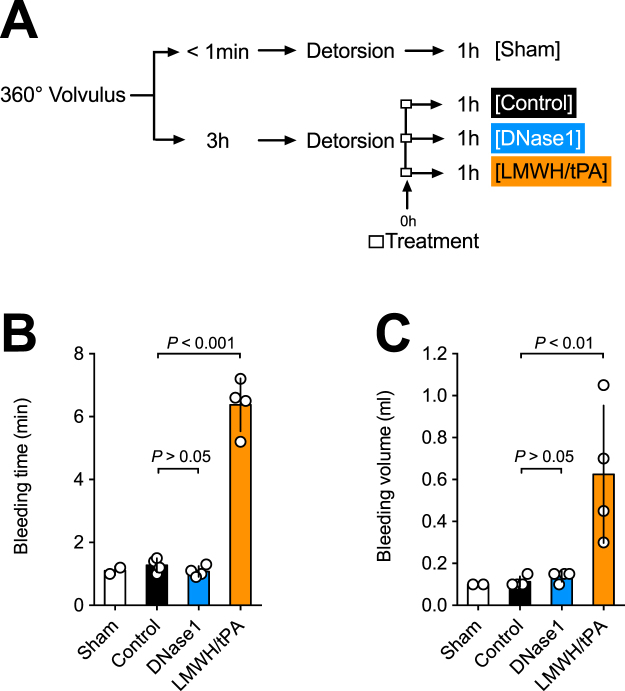
LMWH/tPA, but not DNase1, interferes with hemostasis. (**A**) Experimental design. Rats were subjected to 360° volvulus for <1 min (Sham; N = 2) or 3 hours. Rats received inactivated DNase1 (Control; N = 4), active DNase1 (DNase1, N = 4), or a combination of LMWH and tPA (LMWH/tPA, N = 4) immediately after detorsion of the intestine. One hour after detorsion, blood hemostasis was determined by a tail-bleeding assay. (**B**) Tail bleeding time. (**C**) Loss of blood volume. LMWH/tPA, but DNase1, significantly increased the bleeding time and volume. Data shown as Mean ± SD. Statistics: ANOVA.

Finally, we evaluated the effect of treatment on bleeding time and blood loss. We therefore performed a tail bleeding assay one hour after detorsion and treatment. The time line illustrating the experiment is shown in Fig. 5A. Bleeding time and blood volume were selectively increased in the LMWH/tPA group, while we observed no significant differences amongst controls and the DNase1 treatment group (Fig. 5B). In summary, our study indicates that DNase1 therapy ameliorates intestinal injury and inflammation post midgut volvulus without increasing the risk of bleeding.

## Discussion

The neonatal intestine is highly susceptible to IR injury, thus understanding the pathomechanism of IR injury is crucial^34^. It is known that the initial insult of midgut volvulus is mediated by oxidative stress, as such that the volvulus and consequential oxidative stress compromises the integrity of the endothelial barrier, attracts neutrophils, and promotes thrombosis^35,36^. As newborns have a limited capacity to generate oxidants via xanthine oxidase, oxidative stress is mainly caused by reactive oxygen species (ROS), which are primarily generated by white blood cells, which in turn are recruited to the area of ischemia (i.e. ischemic gut) upon activation of nicotinamide adenine dinucleotide phosphate (NADPH)-oxidase through adhesion or pro-inflammatory cytokines^37^. Our study indicates that induction of midgut volvulus causes both inflammation and thrombosis and that these two factors largely contribute to the devastating consequences of the disease. Treatment with either LMWh/tPA or DNase1 notably reduces intestinal damage after midgut volvulus induction, as seen by significantly reduced levels of apoptosis and oxidative stress in both treatment groups as compared to control group animals.

Moreover, our study’s findings suggest that cfDNA may serve as a diagnostic marker for midgut volvulus. In the current study cfDNA was significantly increased after two, four and six hours post midgut volvulus surgery. The pool of cfDNA is likely made up of DNA of various origins including (1) the formation of NETs^38^ and (2) by passive release of mitochondrial (mtDNA) and nuclear DNA (nDNA) of injured and dying cells^39^. CfDNA might serve as an valuable diagnostic tool to detect intestinal IR injury especially if combined with TAT levels, which indicate hypercoagulation. Similar to cfDNA, TAT levels were significantly increased as early as two hours after midgut volvulus. Consequently, both markers should be evaluated as early predictors of acute mesenteric ischemia in humans.

Similar to IR injury within the central nervous system or liver, intestinal IR injury is characterized by a decreased intestinal barrier function, resulting in a translocation of bacteria followed by an activation of circulating phagocytic cell^40^. Studies show, that i.e. neutrophils are recruited within minutes, with their response peaking at 24–48 hour^41^. Upon activation of the toll like receptor 4 (TLR4), neutrophils are triggered to produce NETs and exacerbate a pro-inflammatory environment^42,43^. As such, it is not surprising that excessive NET-formation may also play a detrimental role in the volvulus pathomechanism, as indicated by the study’s findings. This theory is also supported by a recent study showing that NETs directly cause pulmonary epithelial and endothelial cell damage^44^. Hence targeting NETs with DNase1 to limit inflammation is a logical approach for supportive treatment of any intestinal IR injury. We observed a reduction in circulatory levels of MPO-histone complexes upon DNase1-treatment, indicating toxic effects NETs in our experimental model. However, DNase1 does not specifically target NETs, but degrades extracellular DNA of any source, including damages cells in ischemic tissue. Future studies, employing NET-specific therapies are required to confirm a role of NETs in intestinal infarction.

In addition to the potential role of NETs in the pathogenesis of volvulus, one needs to consider the role of tissue reperfusion and thrombosis. IR injury-induced endothelial cell damage results in a procoagulant and fibrinolysis-suppressing environment, giving rise to an intra- and extravascular fibrin deposition, which further compromises the (micro)circulation of the intestine and promotes necrosis in distal tissue^4^. Mechanisms, that have been proposed to play a role in the procoagulant response are an up-regulation of tissue factors in combination with dysfunctional anticoagulant pathways, along with suppression of fibrinolysis^4^. To assess the role of thrombosis in volvulus, animals were given a combination of enoxaparin (a low molecular weight heparin) and alteplase (a recombinant tissue type plasminogen activator). Heparin is best recognized for its ability to prevent blood coagulation by catalytically accelerating the interaction of antithrombin III with thrombin and factors XIIa, IXa, Vlla, Xa, thereby inhibiting the proteases necessary for completion of the coagulation cascade. In particular, enoxaparin has been shown to have beneficial effects on neutrophil recruitment, oxidative stress, apoptosis and tissue damage, as reported in previous studies^45–47^. In comparison to enoxaparin, alteplase with its fibrinolytic activity, binds to an already formed thrombus and catalyzes the conversion of plasminogen to plasmin. Thus, in this process, the clot is broken down by degradation of fibrin within the matrix of a thrombus. Summing up, alteplase has been shown not only to improve microcirculation, but also to promote neutrophil recruitment to reperfused tissue^48,49^.

Having previously discussed inflammation and thrombosis separately, it should be noted that there is an interrelation of these two factors; inflammation can beget local thrombosis, and thrombosis can amplify inflammation. Especially, in areas of IR injury, platelets colocalize with neutrophils and initiate the release of tissue factors, adhesion molecules, proinflammatory cytokines and prothrombotic microparticles, thus potentiating tissue injury^50^. This interrelation of thrombosis and inflammation suggests a critical role of NETs in IR injury^51^. As demonstrated in the current study, treatment with DNase1 significantly reduced inflammation and thrombosis as measured by decreased parameters of oxidative stress, apoptosis, and tissue damage after intestinal IR injury. In contrast to thrombolysis with alteplase and enoxaparin, DNase1 did not negatively affect coagulation. This is of great importance, as alteplase, in particular, is associated with an increased risk of bleeding complications, such as intracranial hemorrhages in neonates^52^. Further, recent studies have shown that fibrin clots containing DNA and histones have prolonged resistance to fibrinolysis which is reversed by DNase^20^. Consequently, DNase1 treatment after thrombotic events may facilitate thrombolysis. As DNase1 seems to have no relevant side effects, it thus might be suitable for neonates requiring anti-thrombotic treatment. However, systemic DNase1 treatment has only been administered in adult lupus patients and has yet to be studied in neonates^27^. Based on these study’s findings, which show a potential treatment option for volvulus patients, further studies are necessary to examine and validate our results in humans.
